# Inhibitory effect of ribbon-type NF-κB decoy oligodeoxynucleotides on osteoclast induction and activity *in vitro *and *in vivo*

**DOI:** 10.1186/ar1980

**Published:** 2006-07-03

**Authors:** Yasuo Kunugiza, Tetsuya Tomita, Naruya Tomita, Ryuichi Morishita, Hideki Yoshikawa

**Affiliations:** 1 Division of Clinical Gene Therapy, Osaka University Graduate School of Medicine, 2-2 Yamada-oka, Suita, Osaka 565-0871, Japan; 2 Department of Orthopaedics, Osaka University Graduate School of Medicine, 2-2 Yamada-oka, Suita, Osaka 565-0871, Japan; 3 Division of Nephrology, Department of Internal Medicine, Kawasaki Medical School, 577 Matsushima, Kurashiki, Okayama 701-0192, Japan

## Abstract

In this study we examined the effect of ribbon-type (circular-type) NF-κB decoy oligodeoxynucleotides (RNODN) on osteoclast induction and activity. We extracted bone marrow cells from the femurs of rats and incubated non-adherent cells with receptor activator of nuclear factor κB ligand (RANKL) and macrophage colony-stimulating factor (M-CSF). First, transfer efficiency into osteoclasts and their precursors, resistance to exonuclease, and binding activity of decoy to NF-κB were examined. Next, to examine the effect of RNODN on osteoclast induction and activity, osteoclast differentiation and pit formation assays were performed. RNODN were injected into the ankle joints of rats with collagen-induced arthritis. Joint destruction and osteoclast activity were examined by histological study. The resistance of RNODN to exonuclease and their binding activity on NF-κB were both greater than those of phosphorothionated NF-κB decoy oligodeoxynucleotides. The absolute number of multinucleate cells scoring positive for tartrate-resistant acid phosphatase was significantly decreased in the RNODN-treated group. The average calcified matrix resorbed area was significantly decreased in the RNODN-treated group. Histological study showed marked suppression of joint destruction and osteoclast activity by intra-articular injection of RNODN. These results suggest the inhibitory effect of RNODN on the induction and activity of osteoclasts. Direct intra-articular injection of RNODN into the joints may be an effective strategy for the treatment of arthritis.

## Introduction

Osteoclasts are multinucleate giant cells formed by the fusion of hematopoietic cells of the monocyte/macrophage lineage. They are the major resorptive cells of bone [1,2]. In the differentiation pathway of osteoclast progenitors into functionally active osteoclasts, macrophage colony-stimulating factor (M-CSF) is important in proliferation; both M-CSF and receptor activator of NF-κB ligand (RANKL) are essentially involved in differentiation, survival, and fusion; and RANKL enhances osteoclast function [3,4]. The expression of RANKL can be observed in synovial fibroblasts from patients with rheumatoid arthritis (RA) [5]. A crucial target of signaling by RANKL is the activation of NF-κB [6-9]. NF-κB is associated with the activation of osteoclasts and is important in the differentiation of osteoclast precursors [10]. Several studies indicate that selective inhibition of NF-κB in osteoclast precursors prevents osteoclast differentiation and function *in vitro *and *in vivo *[11,12]. Mice deficient in both the p50 and p65 subunits of NF-κB develop osteopetrosis because of a defect in osteoclast differentiation [13,14]. Recently the importance of the IκB kinase (IKK) β subunit as a transducer of signals from RANK to NF-κB for inflammation-induced bone loss and osteoclastogenesis *in vivo *was reported [15].

RA is a chronic inflammatory disease of unknown etiology, characterized by articular inflammation associated with abnormal immune responses and pronounced synovial hyperplasia. Synovial macrophages are capable of differentiating into osteoclasts; the osteoclasts generated within the synovial membrane are probably involved in bone destruction *in vivo *[16]. Multinucleate cells scoring positive for tartrate-resistant acid phosphatase (TRAP) were also induced from CD14-positive cells in the synovial fluid from patients with RA [17]. TRAP-positive multinucleate cells are present in the bone erosion area of patients with RA [18] and also in the bone erosion area of a mouse arthritis model [19,20]. Although the precise mechanism of joint destruction has not been elucidated, osteoclasts seem to have a pivotal role in the joints of patients with RA.

Specific DNA sequences have been used successfully as decoys for binding specific transcription factors, rendering the transcription factors incapable of subsequent binding to the promoter region of target genes [21,22]. This approach has been shown to be effective in modulating gene expression *in vitro *and *in vivo*. The applications of the decoy oligodeoxynucleotides (ODN) strategy against NF-κB have been reported in several studies [23-26]. However, one of the major limitations of the decoy ODN approach is the rapid degradation of phosphodiester ODN by intracellular nucleases. Previously, circular dumbbell double-stranded decoy ODN (we call these ribbon-type decoy ODN) were developed to resolve these issues [27,28]. According to the previous reports, ribbon-type decoy ODN tend to bind more specifically to the transcription factors and have stronger resistance to exonuclease [29,30]. In this study, we tried to use ribbon-type NF-κB decoy ODN for inhibiting the expression of NF-κB, leading to the inhibition of osteoclast induction and activity.

## Materials and methods

### Materials

Ribbon-type decoy ODN and phosphorothionated double-stranded decoy ODN were purchased from Gene Design (Osaka, Japan). Mouse RANKL and mouse M-CSF were purchased from Wako (Tokyo, Japan). Lewis rats were purchased from Clea Japan (Osaka, Japan). Bovine type II collagen was purchased from Cosmo Bio (Tokyo, Japan) and Freund's incomplete adjuvant from Sigma (Munich, Germany).

### Construction of ribbon-type decoy ODN and phosphorothionated double-stranded decoy ODN

**Figure 1:**
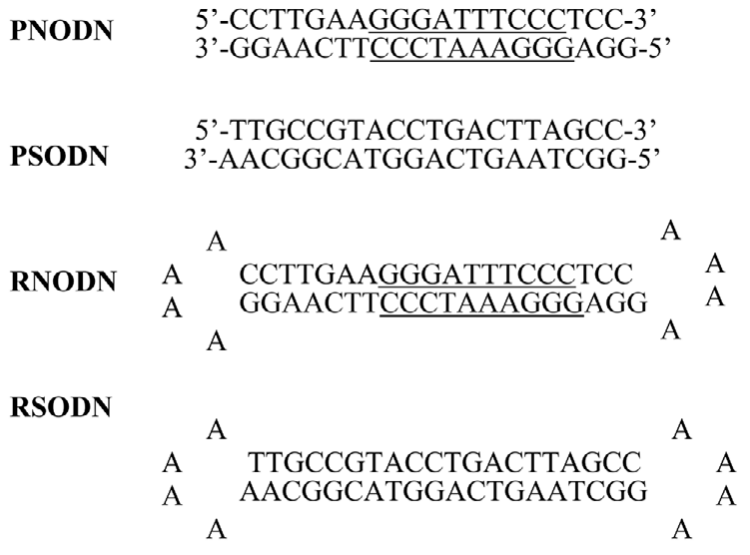
Structures and sequences of the decoy oligodeoxynucleotides used in this study. PNODN and RNODN (phosphorothionated decoy oligodeoxynucleotides) contain the NF-κB-binding site in its double-stranded lesion (consensus sequences are underlined).

The sequences of ribbon-type decoy ODN and phosphorothionated double-stranded decoy ODN are as follows (consensus sequences are shown in bold): ribbon-type NF-κB decoy ODN (RNODN), 5'-TCAAGGAAAACCTTGAA**GGGATTTCCC**TCCAAAAGGA**GGGAAATCCC**T-3' ; ribbon-type scrambled decoy ODN (RSODN), 5'-TAGCCAAAAGGCTAAGTCAGGTACGGCAAAAAATTGCCGTACCTGACT-3' ; phosphorothionated double-stranded NF-κB decoy ODN (PNODN), 5'-CCTTGAA**GGGATTTCCC**TCC-3' and 3'-GGAACTT**CCCTAAAGGG**AGG-5' ; and phosphorothionate double-stranded scrambled decoy ODN (PSODN) 5'-TTGCCGTACCTGACTTAGCC-3' and 3'-AACGGCATGGACTGAATCGG-5' (Figure 1). Decoy ODN containing the NF-κB consensus sequence has been shown to bind the NF-κB transcription factor [24]. PNODN and PSODN were annealed for 2 hours with a steady temperature decrease from 70°C to 25°C. One unit of T4 DNA ligase was added to the mixture, followed by incubation for 24 hours at 22°C to generate a covalently ligated RNODN.

### Resistance to exonuclease

To check resistance for exonuclease, electrophoresis of RNODN and PNODN was performed. In brief, 3 μg of RNODN or PNODN was incubated with exonuclease III at 37°C for 2 hours and then at 65°C for 5 minutes. The solution containing ODN was resolved by electrophoresis on a 19% acrylamide gel.

### Estimation of binding activity

The binding activity of RNODN was examined by using Mercury Transfactor Kits for NF-κB p65 (BD Bioscience, Clontech, Palo Alto, CA, USA) as oligonucleotide competition assays. Kits contain a 96-well format in which wells are coated with an oligonucleotide containing the NF-κB p65 consensus binding sequence. The quantity of nuclear extract binding to the oligonucleotide of the wells is correlated with an increase in signal. An increase in the amount of competitor oligonucleotide corresponds to a decrease in signal because transcription factor binding decreases as the competitor keeps it away from the oligonucleotide-coated surface of the *trans*-Factor well. We estimated the binding activity of oligonucleotides by incubating the same amounts of nuclear protein and various oligonucleotides. An aliquot (30 μg) of TNF-α-stimulated HeLa nuclear extract (Active Motif, Carlsbad, CA, USA) was incubated with decoy ODN (15, 30, and 45 nM) in *trans*-Factor wells for 60 minutes at room temperature. Wells were incubated with primary and secondary antibodies, and the absorbance of the plate was measured with a microplate reader (Model 680; Bio-Rad, Hercules, CA, USA).

### Osteoclast differentiation assay

Bone marrow cells were obtained by flushing femurs of 6-week-old female Lewis rats and were seeded at 2 × 10^7 ^cells per 10 cm Petri dish, then cultured in α-minimal essential medium containing 10% FCS and 1% penicillin/streptomycin. One day after the treatment, non-adherent cells were seeded again and cultured in α-minimal essential medium containing 10% FCS, 1% penicillin/streptomycin, and 20 ng/ml M-CSF in Lab-Tek eight-well chamber slides (Nalge Nunc, New York, NY, USA) at a density of 2 × 10^5 ^cells per well. Two days after the incubation, cells were cultured with 100 ng/ml RANKL and 20 ng/ml M-CSF for 7 days. On days 1, 3, and 5 various decoy ODNs were transiently transferred. Then the cells were washed and stained with a commercial TRAP staining kit (Cell Garage, Tokyo, Japan). The number of TRAP-positive multinuclear (three or more nuclei) cells was counted.

### Pit formation assay

To examine the effect of RNODN on resorbing activity, cells were cultured on BD BioCoat osteologic calcium hydroxyapatite-coated slides (BD Biosciences, Bedford, MA, USA) in a 5% CO_2 _incubator. The non-adherent bone marrow cells were seeded at a density of 10^5 ^cells per well. After incubation for 2 days with 20 ng/ml M-CSF, cells were cultured with 100 ng/ml RANKL and 20 ng/ml M-CSF. On days 3, 5, and 7 various decoy ODNs were transiently transferred, and on day 8 cells were washed vigorously and the calcified matrix resorption area on each disc was measured with a Mac SCOPE image analyzer, version 2.51 (Mitani, Fukui, Japan).

### Immunofluorescence staining

Cells were fixed in 4% paraformaldehyde for 20 minutes at 37°C and treated with 0.5% Triton X-100 for 5 minutes. Cells were then blocked for 30 minutes with 2% goat serum/PBS and incubated in 4 μg/ml rabbit polyclonal antibody against NFATc1 (sc-13033; Santa Cruz biotechnology, Santa Cruz, CA, USA) at 4°C for 16 hours and 400 ng/ml Alexa 488 goat anti-rabbit IgG (A-12373; Invitrogen Molecular Probes, Carlsbad, CA, USA) at room temperature for 60 minutes. The density of fluorescence was estimated by calculating the area of fluorescent cells by NIH image software.

### Induction of arthritis by collagen in rats

This experimental study was performed in accordance with the recommendations in the Guide for the Care and Use of Laboratory Animals of National Institutes of Health (NIH). The protocol was approved by the committee on the Ethics of Animal Experiments in Osaka University. Arthritis was induced by collagen with the use of the modified method described by Trentham and colleagues [31]. In brief, 6-week-old female Lewis rats were immunized intradermally with 0.5 mg of bovine type II collagen, which was dissolved in 0.5 ml of 0.1 M acetic acid at 4°C and emulsified in 0.5 ml of cold Freund's incomplete adjuvant. On day 7, the rats received an intradermal booster injection of half the volume of the first immunization. Onset of arthritis in the ankle joints could be usually recognized visually between days 10 and 14. All rats in which the onset of arthritis could not be recognized visually by day 14 were excluded from this study.

### *In vivo *transfer of fluorescein isothiocyanate (FITC)-labeled RNODN

To examine the localization of RNODN delivery, 50 μg of FITC-labeled RNODN were injected intra-articularly. One day after transfer, synovial tissues were extracted and fixed. Cryostat sections of synovial cells were observed by ultraviolet microscopy (T6300; Nikon, Tokyo, Japan). The sections were also stained with 4',6-diamidino-2-phenylindole.

### Experimental protocol

On day 14 after immunization, 50 μl of suspension containing 200 μg of RNODN or 200 μg of RSODN or 50 μl of PBS was administered intra-articularly with a 30-gauge needle into the right side hind-ankle joint of rats with collagen-induced arthritis (CIA). Administration was performed once every week for 3 weeks. At the end of the experiment (day 35), the ankle joints were fixed in 4% paraformaldehyde, decalcified with EDTA, and embedded in paraffin; sections 4 μm thick were prepared. Next, sections were stained with hematoxylin and eosin. The extent of arthritis in the ankle joints was assessed in accordance with the method reported previously [32]: 0 = normal synovium, 1 = synovial membrane hypertrophy, 2 = pannus and cartilage erosion, 3 = major erosion of cartilage and subchondral bone, and 4 = loss of joint integrity and ankylosis. To investigate the osteoclastic activity *in vivo*, sections were stained with a TRAP staining kit (Cell Garage, Tokyo, Japan). TRAP-positive multinuclear cells were counted in the sections of each ankle (at × 100 magnification). All procedures complied with the standards described in the Osaka University Medical School Guidelines for the Care and Use of Laboratory Animals.

### Statistical analysis

Statistical analysis was performed with the unpaired *t *test and the Mann-Whitney *U *test; *p *< 0.05 was considered significant. All experiments *in vitro *were performed at least three times.

## Results

### Stability of RNODN

**Figure 2:**
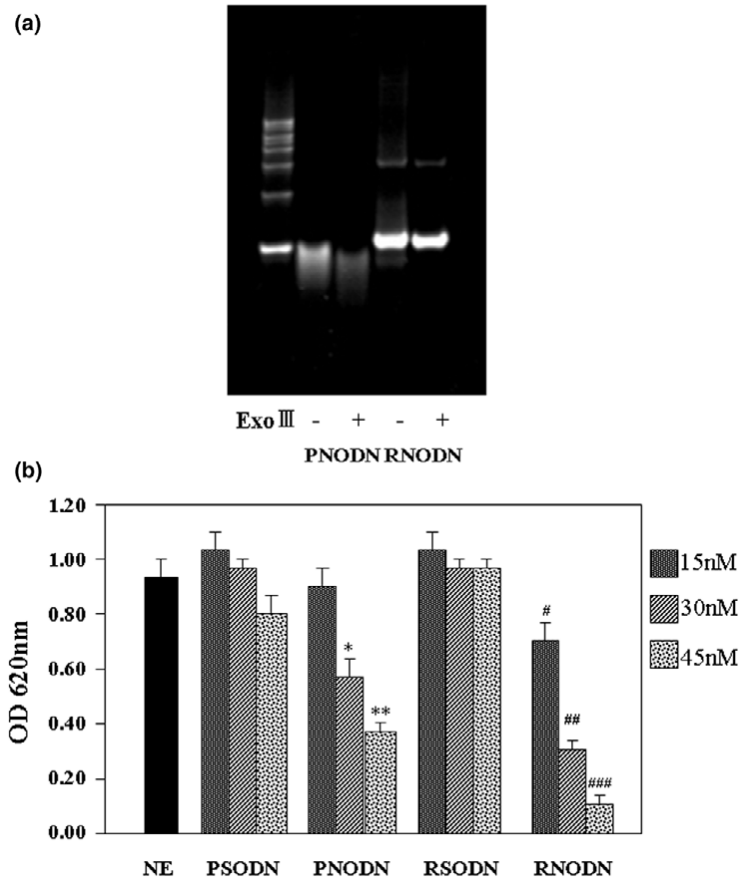
The stability and binding activity of RNODN and PNODN. **(a) **Stability of phosphorothionate double-stranded NF-κB decoy oligodeoxynucleotide (PNODN) and ribbon-type NF-κB decoy oligodeoxynucleotide (RNODN) in the presence of exonuclease III. **(b) **Effects of various decoys on binding activity towards NF-κB. The binding activity of decoy oligodeoxynucleotides (ODNs) reflected their ability to decrease absorbance. NE, nuclear extract without treatment of decoy ODN. (*n *= 5 per group; *p < 0.05, **p < 0.01, ^#^*p *< 0.05, ^##^*p *< 0.01, ^###^*p *< 0.001 compared with nuclear extract without treatment of decoy ODN.)

In this study we used RNODNs to improve stability to exonuclease. Initially, the structural stability of decoy ODN was examined by the ability to resist degradation in the presence of exonuclease III. The primary cause of degradation of standard DNA oligomers in biological applications is a 3'-exonuclease activity found in cells [33,34]. RNODN showed high resistance to exonuclease III and was observed as a major band in gel electrophoresis. In comparison with RNODN, PNODN was degraded after incubation in the presence of exonuclease III (Figure 2a).

### Binding activity of RNODNs on NF-κB

To examine the binding activity of RNODN on the NF-κB protein, an *in vitro *competition assay was performed with Mercury Transfactor Kits for NF-κB p65 (Figure 2b). An increase in the concentration of unbound NF-κB protein was accompanied by a corresponding increase in absorbance. The binding activity of decoy ODNs reflected their ability to decrease the absorbance level. The result of calculating the absorbance of each group is shown as a percentage over that of the untreated group. When PNODN or RNODN was used as a competitor oligonucleotide at 30 or 45 nM, a significant decrease in absorbance was confirmed against the absorbance of nuclear extract without competitor oligonucleotides. A stronger competitive effect was observed when RNODN was used than with PNODN. At a concentration of 15 nM, the competitive effect was observed only in the RNODN-treated group. When RSODN or PSODN was used as a competitor oligonucleotide, the decrease in absorbance was minimal compared with that of nuclear extract without competitor oligonucleotides. The result shows that RNODN has specific and strong binding activity on the NF-κB protein.

### RNODN inhibits RANKL-induced osteoclastogenesis

**Figure 3:**
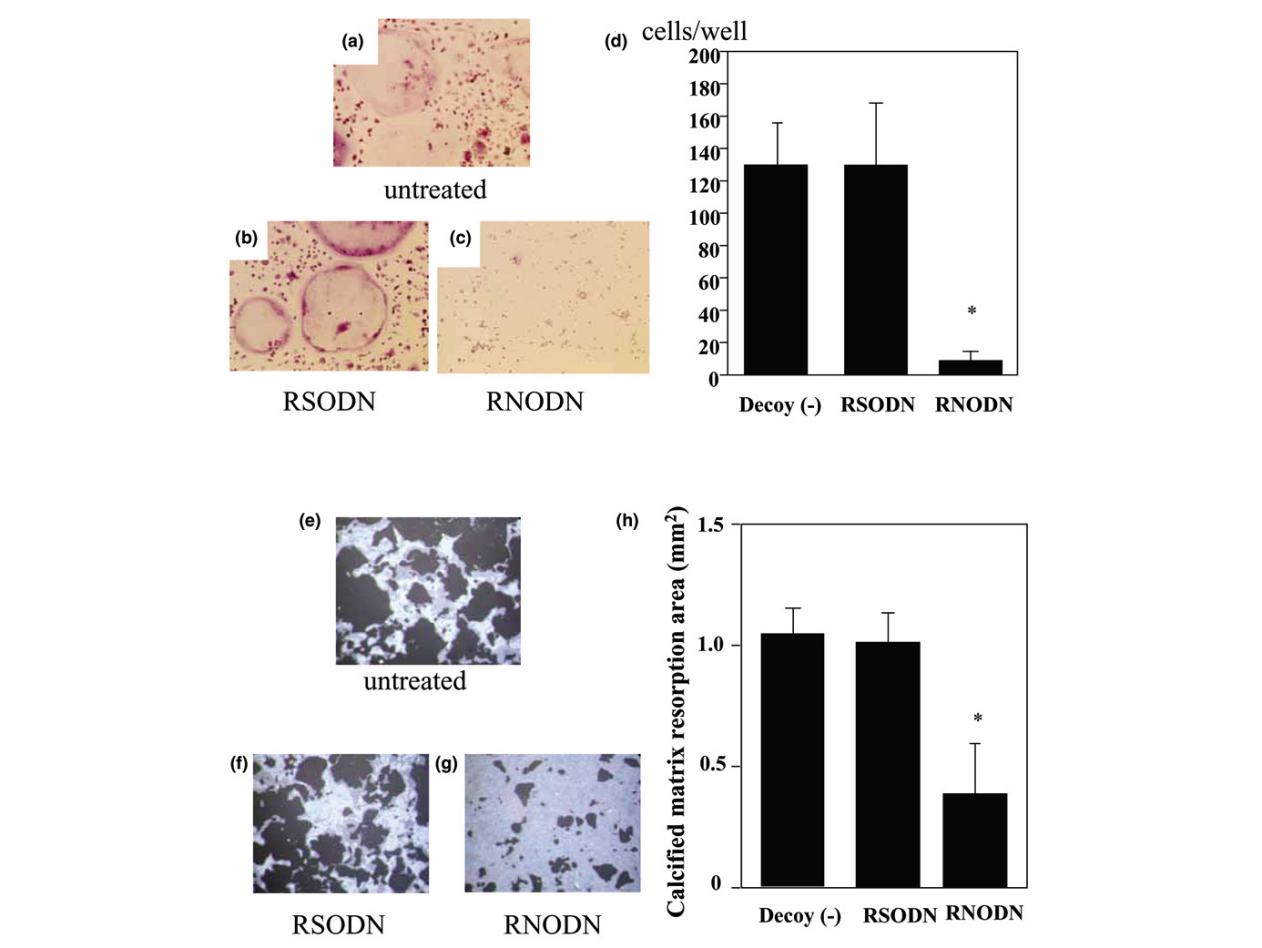
Osteoclast differentiation induced *in vitro *by macrophage colony-stimulating factor and RANKL. Cells were transiently transferred with ribbon-type scrambled decoy oligodeoxynucleotide (RSODN) **(b) **or ribbon-type NF-κB decoy oligodeoxynucleotide (RNODN) **(c)**, or were untreated alone **(a)**. Original magnification × 100. **(d) **Numbers of TRAP-positive multinuclear cells. (*n *= 5 per group; *p < 0.001, compared with rats treated with RSODN.) **(e-h) **Calcified matrix resorption by osteoclast-like cells induced by soluble receptor activator of nuclear factor κB ligand (RANKL). Cells were transiently transferred with RSODN **(f) **or RNODN **(g) **or were untreated alone **(e)**. **(h) **Mean calcified matrix resorption areas calculated by MacSCOPE image analyzer. (*n *= 5 per group; *p < 0.01 compared with the RSODN-treated group.)

To examine the effects of RNODN on osteoclastogenesis *in vitro*, bone marrow macrophages were incubated with decoy in the presence of RANKL and M-CSF (Figure 3a–c). The number of TRAP-positive multinuclear cells in the untreated group and in the RSODN-treated and RNODN-treated groups were 124.2 ± 34.6, 126.2 ± 45.5, and 5.2 ± 1.9, respectively (mean ± SD; Figure 3d). Osteoclastogenesis induced by RANKL was inhibited by incubation with RNODN (*p *< 0.001 compared with the RSODN-treated group). The inhibitory effect was not observed when cells were incubated with RSODN (Figure 3).

### RNODN inhibits RANKL-induced pit formation

To examine the inhibitory effects of RNODN on the activation of osteoclasts, a pit formation assay was performed (Figure 3e–g). The calcified matrix resorption area in the untreated group and in the RSODN-treated and RNODN-treated groups were 1.03 ± 0.12, 1.01 ± 0.12, and 0.36 ± 0.21 mm^2^, respectively (mean ± SD; Figure 3h). Results showed that calcified matrix resorption by RANKL-induced osteoclast-like cells was significantly inhibited by incubation with RNODN (*p *< 0.01 compared with the RSODN-treated group). The inhibitory effect was not observed when cells were incubated with RSODN.

### Downregulation of NFATc1 by RNODN

**Figure 4:**
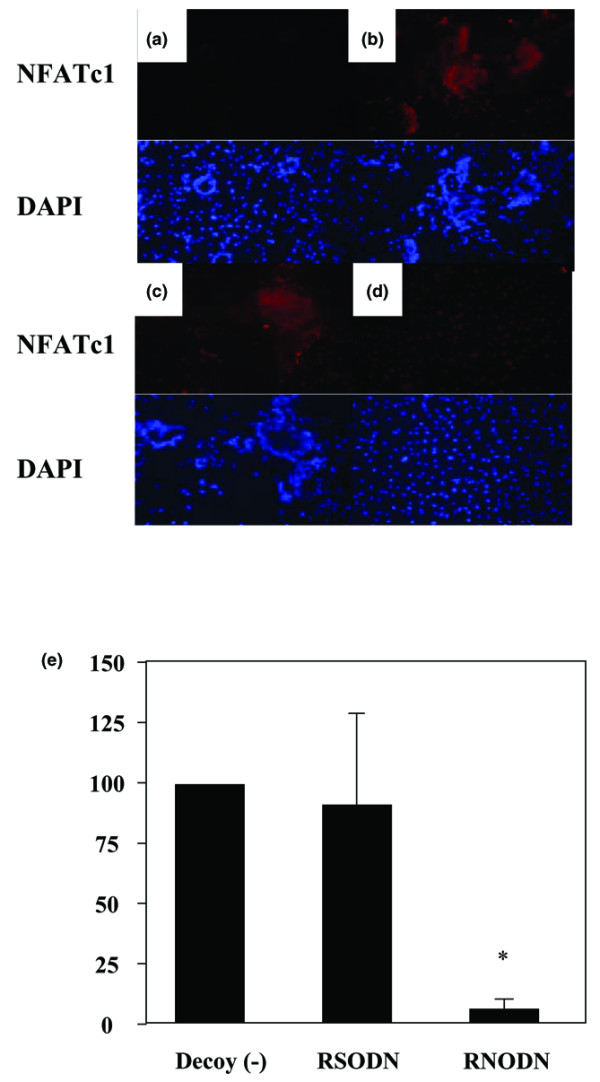
Expression of NFATc1 protein in osteoclast precursor cells. **(a-d) **Immunohistochemistry of NFATc1 protein with specific antibody in osteoclast precursor cells. Bone marrow macrophages were incubated with M-CSF/RANKL for 48 hours after incubation with ribbon-type scrambled decoy oligodeoxynucleotide (RSODN) (c) or ribbon-type NF-κB decoy oligodeoxynucleotide (RNODN) (d), or were untreated alone (b). (a) Without reaction with primary antibody. The expression of NFATc1 by immunofluorescence is shown in each upper panel. Nuclei stained with 4',6-diamidino-2-phenylindole are shown in each lower panel. Original magnification × 100. **(e) **Measurement of fluorescent area of osteoclast precursor cells. The areas of fluorescent cells in RSODN-treated and RNODN-treated groups are shown as percentages over that of the untreated group. (*n *= 5 per group; *p < 0.001 compared with the RSODN-treated group.)

To clarify the mechanism underlying the inhibitory effect of RNODN on osteoclastogenesis, we examined the expression of the NFATc1 protein in bone marrow cells incubated with RANKL. NFATc1 is a master switch for regulating the terminal differentiation of osteoclasts, functioning downstream of RANKL [35]. As shown in Figure 4, the expression of NFATc1 in RANKL-stimulated bone marrow cells increased in accordance with the fusion of cells (Figure 4b). The results of calculating the area of fluorescent cells in RSODN-treated and RNODN-treated groups are shown as percentages over that of the untreated group. The data for each group (mean ± SD) are 90.0 ± 38.6% and 3.5 ± 3.2%, respectively (Figure 4e). The expression of NFATc1 was inhibited by incubation with RNODN (*p *< 0.001 compared with the RSODN-treated group; Figure 4d).

### *In vivo *transfer of FITC-labeled RNODN into joint synovium

**Figure 5:**
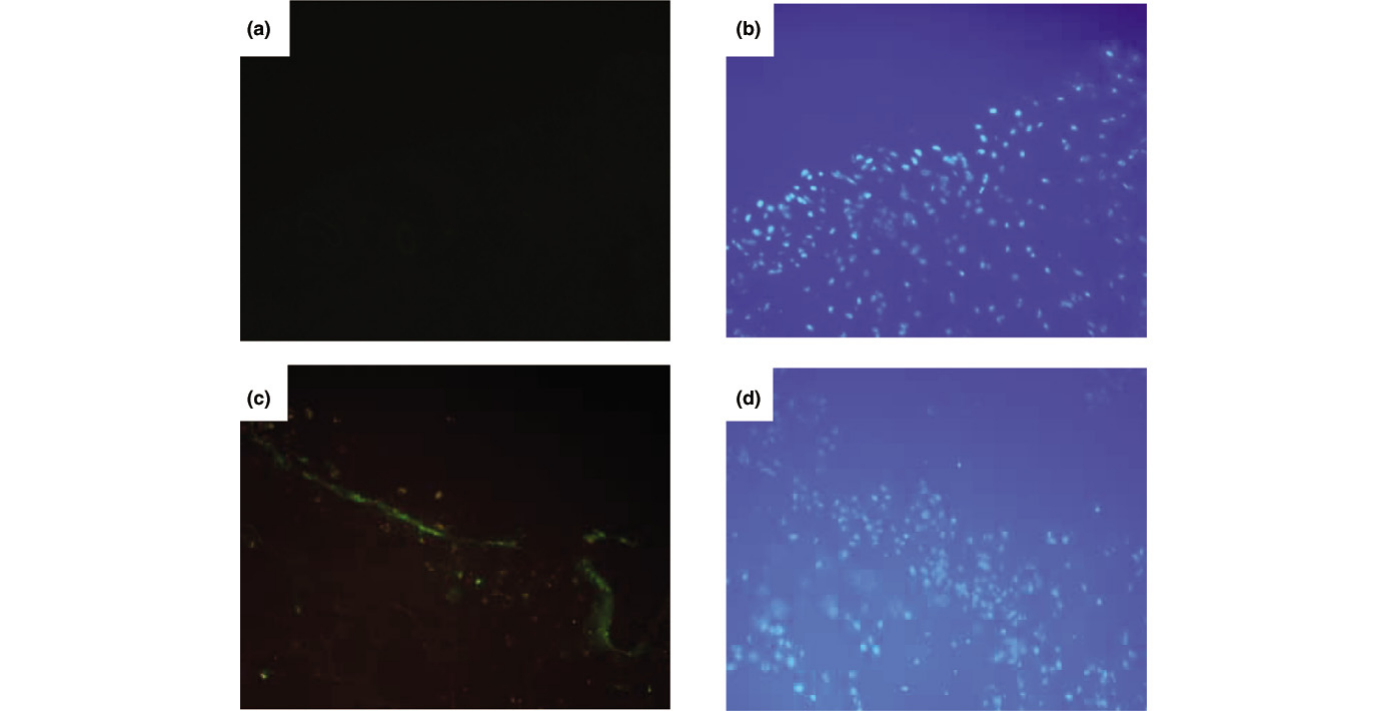
Representative findings of fluorescence microscopy of synovium transferred with FITC-labeled RNODN. **(a) **Synovium transferred with ribbon-type NF-κB decoy oligodeoxynucleotide (RNODN) not labeled with fluorescein isothiocyanate (FITC). **(b) **The sections were counterstained with 4',6-diamidino-2-phenylindole. **(c) **Synovium transferred with FITC-labeled RNODN. Original magnification × 200. **(d) **The sections were counterstained with 4',6-diamidino-2-phenylindole.

We performed *in vivo *transfer of FITC-labeled RNODN into rat ankle joints. Fluorescence was localized in synovial cells, especially the surface area (Figure 5c). Synovium transferred with decoy ODN not labeled with FITC showed no specific fluorescence (Figure 5a). The nucleus was stained with 4',6-diamidino-2-phenylindole (Figure 5b,d).

### Inhibitory effects of intra-articular injection of RNODN on joint destruction and osteoclast activity in rats with CIA

**Figure 6:**
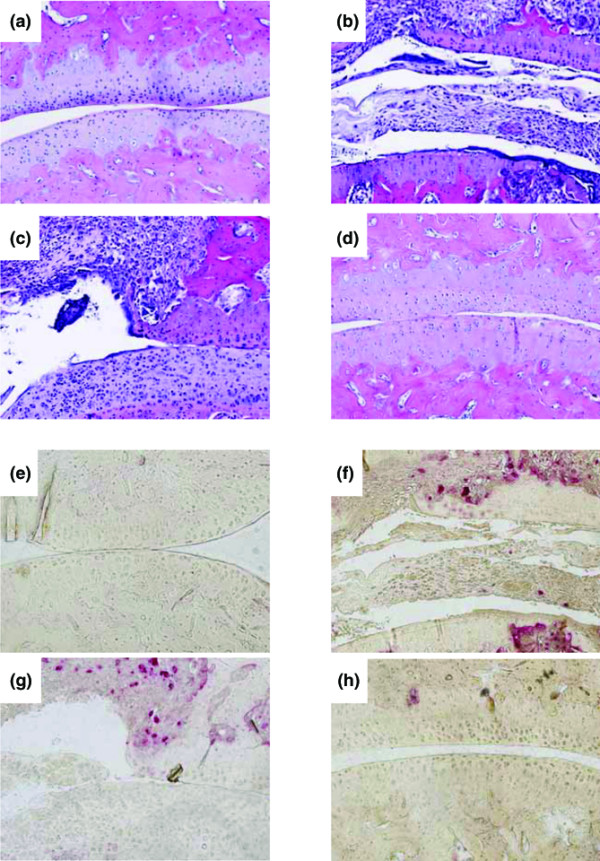
Histological analysis in the ankle joints of rats with collagen-induced arthritis (CIA) at day 35. Samples were stained with hematoxylin and eosin in **(a-d) **and with tartrate-resistant acid phosphatase [TRAP] in **(e-h)**. (a) Naive rats had normal joints. (b) Ankle joints of rats with CIA treated with PBS showed pannus invasion and massive cellular infiltration of the synovium, with disruption of cartilage and subchondral bone. (c) Ankle joints of rats with CIA treated with ribbon-type scrambled decoy oligodeoxynucleotide (RSODN) also showed pannus invasion and massive cellular infiltration of the synovium, with disruption of cartilage and subchondral bone. (d) Ankle joints of rats with CIA treated with ribbon-type NF-κB decoy oligodeoxynucleotide (RNODN) also had nearly intact articular joints. (e) Ankle joints of naive rats had few TRAP-positive multinuclear cells. (f) Ankle joints of rats with CIA treated with PBS showed active resorption of cartilage and subchondral bone by pannus and synovium including TRAP-positive multinuclear cells. (g) Ankle joints of rats with CIA treated with RSODN also showed active resorption of cartilage and subchondral bone by pannus and synovium including TRAP-positive multinuclear cells. (h) TRAP-positive multinuclear cell formation was suppressed in ankle joints of rats with CIA treated with RNODN. Original magnifications × 100.

To evaluate the effect of RNODN on joint destruction and osteoclast activation, we performed a histological analysis of the ankle joints treated with RNODN, RSODN, or PBS. Histologically, ankle joints of rats with CIA treated with PBS (Figure 6b) or RSODN (Figure 6c) showed pannus invasion and massive cellular infiltration of the synovium, with disruption of cartilage and subchondral bone. Conversely, ankle joints of rats with CIA treated with RNODN (Figure 6d) showed marked improvement in arthritis. The arthritis scores (mean ± SD) of PBS-treated joints, RSODN-treated joints, and RNODN-treated joints were 3.0 ± 0.7, 3.2 ± 0.8, and 1.8 ± 0.8, respectively (Table 1). The number of osteoclasts around the ankle joints was significantly smaller in RNODN-treated rats than in RSODN-treated or PBS-treated rats (Figure 6f,g). The numbers of osteoclasts in PBS-treated joints, RSODN-treated joints, and RNODN-treated joints were 142.8 ± 15.1, 153.8 ± 28.2, and 31.0 ± 27.3, respectively (Table 1). Figure 6a and Figure 6e show HE staining and TRAP staining of ankle joints in naive rats.

## Discussion

The Rel/NF-κB family of transcription factors is induced in response to several signals. In unstimulated cells, NF-κB is associated in the cytoplasm with the inhibitory protein IκB. In response to an external signal, IκB is phosphorylated and degraded, releasing NF-κB to enter the nucleus and activate transcription [36,37]. The wide variety of genes regulated by NF-κB includes cytokines, chemokines, adhesion molecules, acute-phase proteins, and inducible effector enzymes. The important role of NF-κB in the differentiation and activation of osteoclasts has been mentioned previously [38]. Selective inhibition of NF-κB by several drugs blocks osteoclastogenesis [11,12]. In the present study we have shown that selective inhibition of NF-κB with a ribbon-type NF-κB decoy could suppress the differentiation and activation of RANKL-induced osteoclastogenesis. Transfection of decoy ODN corresponding to the *cis *sequences result in the attenuation of authentic cis-trans interaction, leading to the removal of *trans*-factors from the endogenous *cis*-element, with subsequent modulation of gene expression [39]. The principle of the transcription factor decoy approach is based on the reduction of promoter activity as a result of the inhibition of binding of a transcription factor to a specific sequence in the promoter region. This approach is relatively simple and can be targeted to specific tissues; decoy ODN can be more effective than antisense ODN in blocking constitutively expressed factors as well as multiple transcription factors that bind to the same *cis *element [39]. However, one of the major limitations of the decoy ODN approach is the rapid degradation of phosphodiester ODN by intracellular nucleases [40-42]. The lack of sequence specificity of phosphodiester ODN has been reported previously [29,43,44]. To overcome these issues, the circular dumbbell double-stranded decoy ODN was developed [42,45,46]. Circular dumbbell decoy ODN for AP-1 or E2F have been demonstrated to be more effective than conventional decoy ODN in previous studies [40,41]. In this study, RNODN showed higher resistance to exonuclease and stronger binding activity on NF-κB than PNODN, and we examined the effect of RNODN for the inhibition of osteoclast differentiation and activation. A previous report [47] showed the effect of decoy targeting NF-κB on apoptosis of human osteoclasts. In contrast to their results we were unable to show the specific effect of RNODN for apoptosis of rat osteoclasts. It is not yet clear whether NF-κB is responsible for the survival of osteoclasts [48].

In this study, we were able to transfer decoy ODN to adherent macrophage/monocyte-like cells and osteoclast-like cells without reagent. The possibility and effectiveness of ODN transfer into these cells have been reported previously [49]. The cellular uptake of ODN is reportedly achieved by a receptor-mediated endocytosis mechanism [50,51]. However, the exact mechanism of cellular uptake of naked DNA or ODN is still poorly defined [52]. The efficiency of internalizing naked DNA varies between cell types [52]. In our study, the effectiveness of ODN transfer was promoted in serum-free conditions. The size of the ribbon-type decoy is about 20 base pairs, which is small compared with the plasmid, so it may be easier for ODN to be transferred into osteoclasts or their precursors.

In the pit formation assay of this study, we transferred the decoy on day 3. We were able to confirm TRAP-positive multinuclear cells on day 3 but the cells were not so large and it might be difficult to state that these cells were mature osteoclasts. It would have been better if we could have incubated mature osteoclasts on a hydroxyapatite-coated disc, but osteoclasts are easily damaged and it is technically difficult to subculture rat mature osteoclasts.

In the previous study, the gene encoding NFATc1, a member of the NFAT family of transcription factor genes, was found to be the most strongly induced transcription factor gene after stimulation by RANKL in osteoclast differentiation. NFATc1 autoamplifies its own gene, possibly by binding to its own promoter [35]. The AP-1 and NF-κB binding sites are present with the promoter region of the NFATc1 gene [53]. Recently, Takatsuna and colleagues showed that (-)-DHMEQ, a newly designed NF-κB inhibitor, inhibited RANKL-induced osteoclast differentiation in mouse bone marrow macrophages through the downregulation of NFATc1 [54]. In the present study the expression of NFATc1 was inhibited by treatment with RNODN.

The skeletal complications of RA consist of focal bone erosions and periarticular osteoporosis at sites of active inflammation, and generalized bone loss with reduced bone mass. In rheumatoid synovium, activated T cells and fibroblasts express RANKL. TNF-α and IL-1β are also overproduced in synovium. TNF-α and IL-1 β, acting in concert with RANKL, can powerfully promote osteoclast recruitment, activation, and osteolysis in RA [55]. In the synovium of patients with RA, NF-κB was present in most macrophages within the lining and sublining lesions throughout the synovium, including endothelial cells [56,57]. CIA is an autoimmune model that in many ways resembles RA. Immunization of genetically susceptible rodents with type II collagen leads to the development of severe polyarticular arthritis mediated by an autoimmune response. Just as in RA, synovitis and erosions of cartilage and bone are hallmarks of CIA [58]. In the present study, direct injection of RNODN in arthritic joints of rats with CIA led to an amelioration of arthritis and decreased the number of TRAP-positive cells in the synovium. The strategy of naked RNODN transfer into the joint implies a potential for future clinical treatment.

## Conclusion

RNODN showed higher resistance to exonuclease and higher binding activity on NF-κB than did PNODN. Differentiation and calcium resorption were suppressed by treatment with RNODN, by preventing NFATc1 expression. Joint destruction and osteoclast activity were significantly suppressed by intra-articular injection of RNODN.

These data suggest that RNODNs inhibit the induction and activity of osteoclasts and that the direct injection of RNODNs into the joints might be an effective strategy for the treatment of arthritis.

## Abbreviations

FCS = fetal calf serum; FITC = fluorescein isothiocyanate; IL = interleukin; M-CSF = macrophage colony-stimulating factor; NF-κB = nuclear factor-κB; ODN = oligodeoxynucleotide; PBS = phosphate-buffered saline; PNODN = phosphorothionate double-stranded NF-κB decoy ODN; PSODN = phosphorothionate double-stranded scrambled decoy ODN; RA = rheumatoid arthritis; RANKL = receptor activator of NF-κB ligand; RNODN = ribbon-type NF-κB decoy ODN; RSODN = ribbon-type scrambled decoy ODN; TNF = tumor necrosis factor; TRAP = tartrate-resistant acid phosphatase.

## Competing interests

The authors declare that they have no competing interests.

## Authors' contributions

YK performed molecular and animal experiments, measurements and evaluation of the data, and statistical analyses. TT supervised the study design, the interpretation of data, and the writing of the manuscript. TN conceived and participated in the experimental design of the study. RM and HY supervised the study design and gave valuable advice to YK. All authors read and approved the final manuscript.

## Supplementary Material

Additional File 1 A PDF containing a supplementary figure that demonstrates that there is no activity in the nuclear extracts leading to time-dependent degradation of DNA. Click here for file

Additional File 2 A PDF containing a supplementary figure that examines the effects of RSODN and RNODN on cell growth. Click here for file

## Figures and Tables

**Table 1:** Mean histological scores and osteoclast numbers of rats with collagen-induced arthritis

Group	Number of joints	Histological score	Osteoclast number
PBS injection	5	3.0 ± 0.7	142.8 ± 15.1
RSODN injection	5	3.2 ± 0.8	153.8 ± 28.2
RNODN injection	5	1.8 ± 0.8^a^	31.0 ± 37.3^b^

^a^*p *< 0.01 compared with PBS injection group; ^b^*p *< 0.01 compared with PBS-injection group (*n *= 5 rats and *n *= 5 joints for each group). RNODN, ribbon-type NF-κB decoy oligodeoxynucleotide; RSODN, ribbon-type scrambled decoy oligodeoxynucleotide. Results are means ± SD.
