# Supplementary figures and images for: Inhibitory effect of ribbon-type NF-κB decoy oligodeoxynucleotides on osteoclast induction and activity in vitro and in vivo
Source: Arthritis Res Ther. 2006 Jul 3;8(4):R103. doi: 10.1186/ar1980 (PMC1779370; doi:10.1186/ar1980)

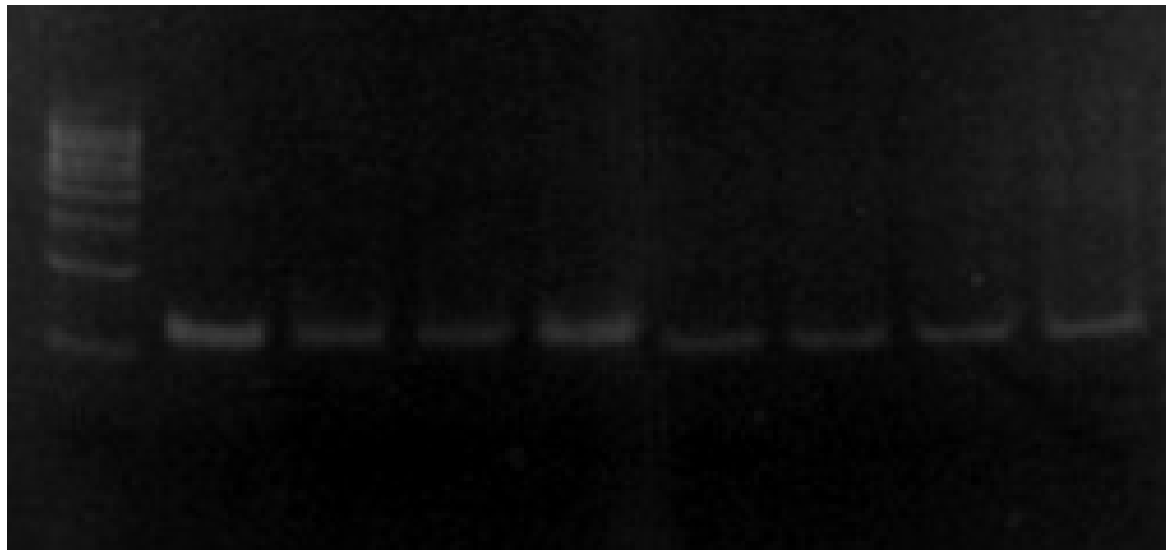

0 0.5 1 2 0 0.5 1 2  
(h) (h)  
PNODN RNODN

**Sup Figure 1.** Kunugiza Y et.al.

Supplement: Additional File 1 — A PDF containing a supplementary figure that demonstrates that there is no activity in the nuclear extracts leading to time-dependent degradation of DNA. [file ar1980-S1.pdf]

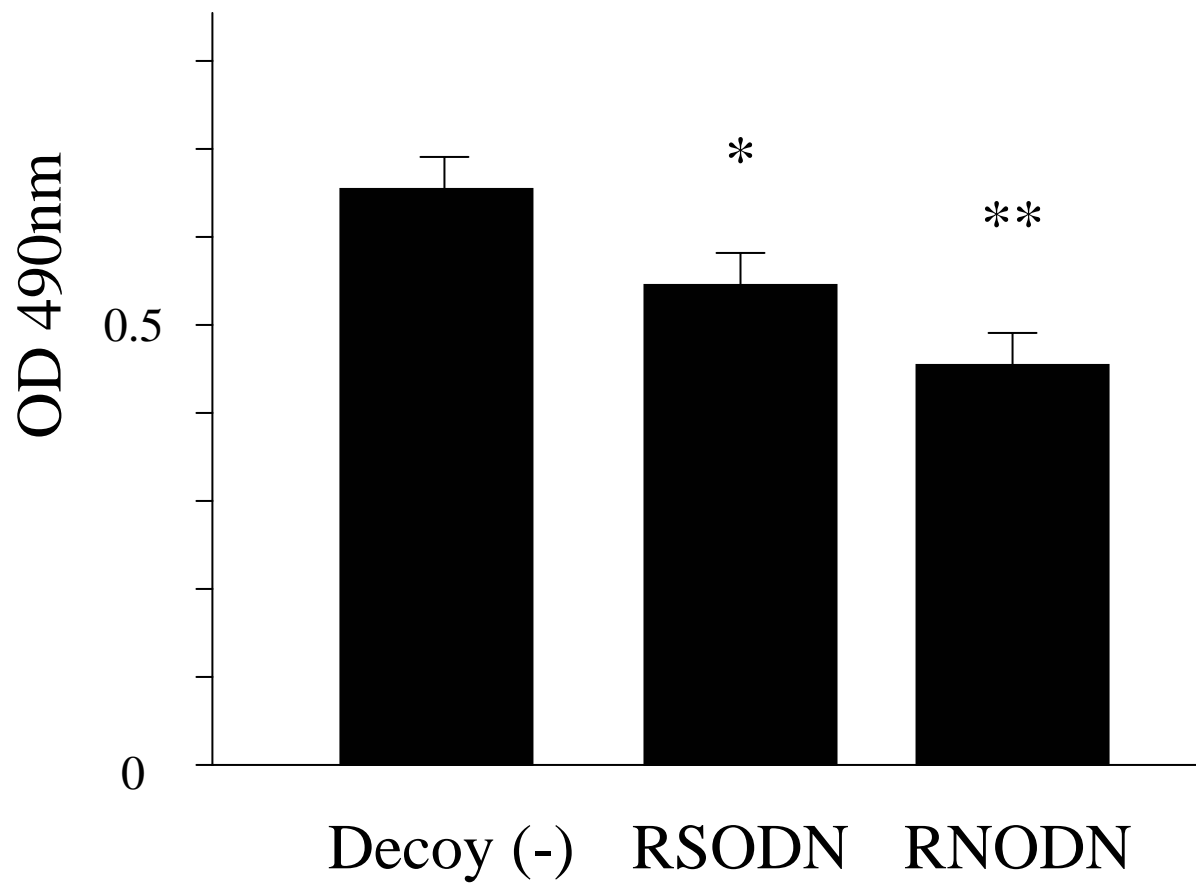

**Sup Figure 2.** Kunugiza Y et.al

Supplement: Additional File 2 — A PDF containing a supplementary figure that examines the effects of RSODN and RNODN on cell growth. [file ar1980-S2.pdf]
